# Sensorimotor integration in *Caenorhabditis elegans*: a reappraisal towards dynamic and distributed computations

**DOI:** 10.1098/rstb.2017.0371

**Published:** 2018-09-10

**Authors:** Harris S. Kaplan, Annika L.A. Nichols, Manuel Zimmer

**Affiliations:** Research Institute of Molecular Pathology, Vienna Biocenter, Campus-Vienna-Biocenter 1, 1030 Vienna, Austria

**Keywords:** *C. elegans*, sensorimotor integration, motor control, whole-brain imaging, mixed selectivity, neuronal population dynamics

## Abstract

The nematode *Caenorhabditis elegans* is a tractable model system to study locomotion, sensory navigation and decision-making. In its natural habitat, it is thought to navigate complex multisensory environments in order to find food and mating partners, while avoiding threats like predators or toxic environments. While research in past decades has shed much light on the functions and mechanisms of selected sensory neurons, we are just at the brink of understanding how sensory information is integrated by interneuron circuits for action selection in the worm. Recent technological advances have enabled whole-brain Ca^2+^ imaging and Ca^2+^ imaging of neuronal activity in freely moving worms. A common principle emerging across multiple studies is that most interneuron activities are tightly coupled to the worm's instantaneous behaviour; notably, these observations encompass neurons receiving direct sensory neuron inputs. The new findings suggest that in the *C. elegans* brain, sensory and motor representations are integrated already at the uppermost sensory processing layers. Moreover, these results challenge a perhaps more intuitive view of sequential feed-forward sensory pathways that converge onto premotor interneurons and motor neurons. We propose that sensorimotor integration occurs rather in a distributed dynamical fashion. In this perspective article, we will explore this view, discuss the challenges and implications of these discoveries on the interpretation and design of neural activity experiments, and discuss possible functions. Furthermore, we will discuss the broader context of similar findings in fruit flies and rodents, which suggest generalizable principles that can be learnt from this amenable nematode model organism.

This article is part of a discussion meeting issue ‘Connectome to behaviour: modelling *C. elegans* at cellular resolution’.

## 1. Introduction

More than three decades ago, the first complete connectome of a nervous system was published in this journal [1]. The *Caenorhabditis elegans* connectome provided some immediate insights into sensorimotor behaviour in the worm [2]. For example, most neurons could be classified as either sensory neurons (those with specialized sensory endings), motor neurons (those with neuromuscular junctions) or interneurons (those lacking both sensory and motor features). Motor neurons could be further subdivided into classes targeting head or body muscles. Five premotor interneurons appeared to be network hubs, receiving a large amount of inputs from sensory circuits and representing a bottleneck for the outputs to body motor neurons.^1^ Many studies have since confirmed a primary role for premotor interneurons in locomotion [3–5]. These immediate insights allowed for one particularly tantalizing prediction: the computational rules underlying sensorimotor behaviour, from single-cue chemotaxis to multisensory integration, must be implemented somewhere in the dense connectivity of interneurons bridging sensory input and motor output.

**Figure 1.**
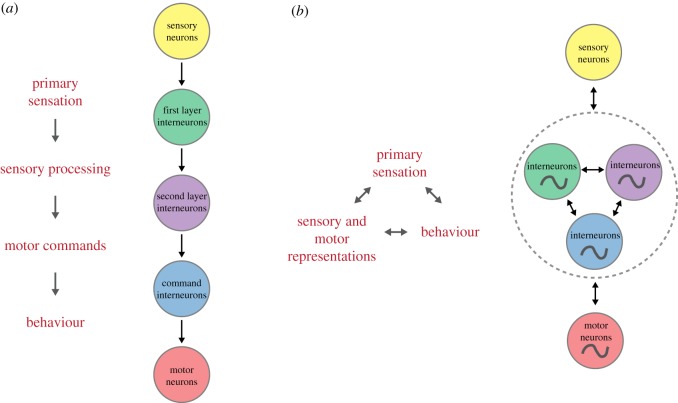
Two contrasting models of sensorimotor flow in *C. elegans*. (*a*) Segregated sequential computations. The transformation from sensory to motor representations is mainly feed-forward and functionally segregated at the neural circuit levels. Computations are performed in a sequential temporal order. (*b*) Distributed computations. More consistent with experimental data is a distributed representation of sensory and motor variables across neuronal circuits. Feedback between most elements is an important property of the system. Inputs are integrated with the internal dynamics of neural circuits (oscillator symbols). Computations could be performed in a concurrent fashion (double arrow heads). This model enables dynamic reciprocal interactions between the brain, body and environment.

Subsequent studies supported the predicted importance of interneurons. Many interneurons were shown, by genetics and laser ablation, to be required for chemotaxis to various sensory cues [6–8]. Depending on the sensory cue, a minimal circuit could be drawn consisting of a handful of neurons and connections [6–9]. For example, a connectome analysis by Gray *et al*. [9] identified two separate layers of interneurons between sensory input and motor output. This suggested a sequential computational strategy in which sensory information from multiple cues is integrated in four first-layer interneurons, which then send some (perhaps transformed) output of the sensory information to three second-layer interneurons; these interneurons finally synapse onto head motor neurons and the premotor hub interneurons to instruct behaviour. This flow of sensorimotor transformation is schematically shown in figure 1*a*. All that remained to be shown was how sensory information, in the form of neural activity, flows through and is transformed by this small group of interneurons.

Such a detailed understanding of sensorimotor behaviour appeared within reach with the advent of calcium imaging more than one decade ago. Results so far, however, have been puzzling. Promisingly, controlled delivery of sensory stimuli to immobilized worms revealed a transfer of olfactory [10–12], gustatory [13,14], thermosensory [15] and gas [16] stimulus information to first-layer interneurons. However, in other studies, calcium imaging in freely moving animals revealed that these same neurons' activities are tightly coupled to the worm's instantaneous behaviour [17–20]. Remarkably, such strong relationships between interneuron activity and instantaneous behaviour were soon observed, by several groups, for all of the aforementioned interneurons. While this relationship was expected for premotor interneurons, both first- and second-layer interneuron activities were also shown to be tightly coupled to various behavioural parameters like direction, speed of locomotion or head posture. Importantly, all these behaviour–activity relationships were found in animals not presented with a sensory stimulus. Collectively, these studies showed that the worm's interneurons do not wait quietly for sensory inputs and subsequently pass the output of their computations onto the next layer. Rather, interneuron activity is extremely dynamic; sensory inputs must somehow be integrated into these ongoing dynamics, in many cases already at the first synapse [19,20].

For the majority of these interneurons, behaviour-correlated activities were shown not to be solely proprioceptive and are therefore best understood as representing motor command signals. This interpretation comes from recordings in immobilized animals, in which interneuron (and motor neuron) activities were dynamic and coordinated among each other, and neurons co-active in these recordings were active in the same motor state in freely moving animals with rare exception [20]. In freely moving animals, activity in these neurons exhibited a nearly one-to-one relationship with behaviour [20]. We therefore refer to all such activity as reflecting motor command signals, which can be generated in immobilized animals presumably in an attempt to move. We further distinguish low-level motor command signals, which directly encode body posture, from high-level motor commands, which rather command longer lasting behavioural states (e.g. forward or reverse locomotion) or movement parameters (e.g. locomotion speed), but do not consist of signals patterned for coordinated muscle contraction. For example, activity of the interneuron RIB is correlated with the animal's forward locomotion speed [18,20]; this information has to be transformed by motor neurons into oscillatory activity controlling the animal's body undulation at the commanded speed. While RIB activity encodes a high-level representation of speed, motor neurons generate the corresponding low-level motor commands. With the exception of one interneuron, RIA, interneuron activities reported thus far correspond to high-level motor command signals.

How, then, do sensory inputs affect ongoing motor-related interneuron dynamics? In the following sections, we will explore this question. We will focus on how sensory inputs bias reversal probability, a fundamental behavioural strategy underlying worm chemotaxis [21]. In box 1 and box 2, we illuminate how these recent findings affect experimental design and the interpretation of results. In the next section, we integrate findings in the recent literature that call for an updated model for sensorimotor computations in *C. elegans*. We then discuss new non-mutually exclusive hypotheses raised by these recent discoveries. Specifically, we suggest that these motor representations could serve sensory gain control by behavioural state and/or forward internal models for precise motor control. We also put these findings in the larger context of sensorimotor integration in other species. Similar ‘early’ representations of motor state have been found in fruit flies and in mammalian cortical primary sensory areas. Combined with the discovery of population dynamics in the recurrent network underlying *C. elegans* behaviour, these observations suggest a great potential to learn generalizable principles from this model organism.

Box 1. **Interneuron activity is more strongly correlated to behaviour than to sensory input.**

The finding that many interneurons' activities show tight coupling to the worm's instantaneous motor state has major implications for interpreting sensory responses. Here, we illustrate these implications using AVA interneuron activity and O_2_ shifts as an example. AVA activity was recorded in freely moving animals during six consecutive 30 s switches between 4% O_2_ and 21% O_2_ [20]. During high O_2_ periods, animals reverse more than during low O_2_ periods [20]. Trial averaging AVA responses across stimuli and worms revealed a rise in AVA activity upon O_2_ upshift (*a*, average ± STD, *n* = 36 shifts across six animals). This suggests that AVA is part of the sensorimotor pathway leading to reversal frequency modulation by this stimulus. However, AVA shows spontaneous activity changes in both freely moving and immobilized animals regardless of O_2_ levels [20]; in moving worms, AVA activity rises correlate exclusively and reliably with reversal events [4,20,23–26]. Indeed, trigger averaging AVA activity to reversal onset during the 21% O_2_ period (*b*, grey shows 10th–90th percentiles, *n* = 136 reversals from six animals) revealed a much stronger modulation of AVA activity by behaviour than by sensory stimulus (compare *a* and *b*, same *y*-axis scale).

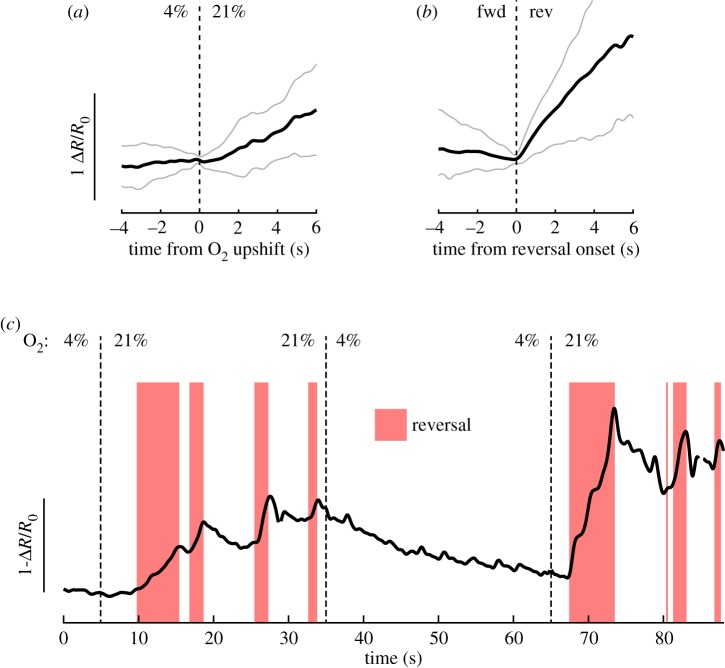

An example trace in (*c*) shows two consecutive O_2_ shifts (*t* = 5 s and *t* = 65 s). Here, we see that the trial average in (*a*) is misleading: in single trials, reversal neurons such as AVA do not show a long-lasting rise in activity but rather an increased probability of high activity states, corresponding to the increased probability of reversals. Furthermore, AVA upshift response varies in onset timing, slope, amplitude and secondary peak number; all of this variation is explained by variation in reversal behaviour events [20]. Still, O_2_ levels modulate reversal probability, such that, on average, an AVA rise in response to O_2_ upshift can be observed. Because many interneurons show similarly tight behaviour coupling (see the main text), the pitfalls in this example hold true across other interneurons and stimuli. Recordings in immobilized animals are even more susceptible to these caveats due to the lack of concurrent behaviour recording and longer timescales of spontaneous activity changes (see box 2).

Box 2. **Analysing interneuron activity responses to sensory stimulation.**

Recent findings from several laboratories have shown that the vast majority of interneurons exhibit spontaneous activity changes in the absence of acute sensory inputs, in both freely moving and immobilized animals. If interneuron activity is dynamic, how do we assess the impact of sensory stimulation on these ongoing activities? Here, we propose three steps that should be taken to analyse such data. We go through these steps with an example, ultimately showing that they can lead to clearer, more mechanistic interpretations of interneuron sensory responses. We focus on a single interneuron and stimulus, but these steps generally suffice for most interneurons and stimulus paradigms.
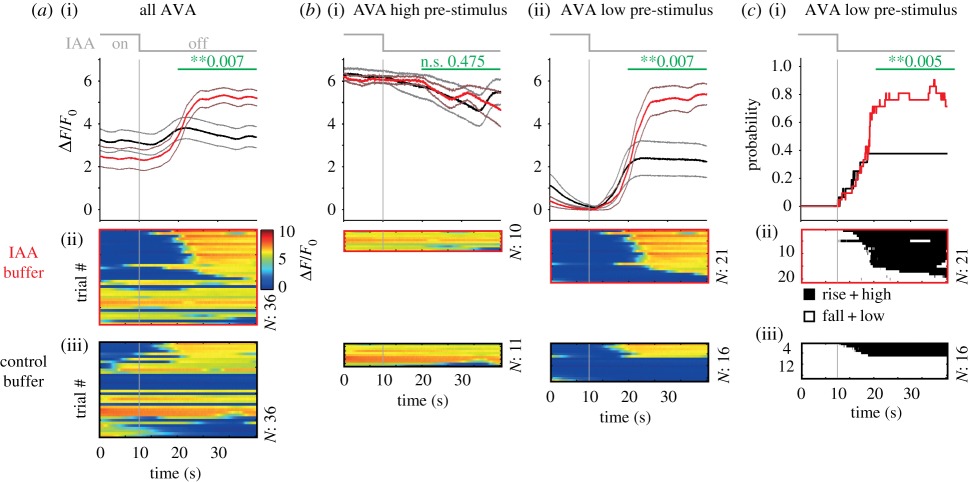

(*a*) Step 1: Record long pre-stimulus periods to characterize spontaneous activity changes and pre-stimulus activity state.

We recorded GCaMP signals from interneuron AVA from each animal for a total of 12 min. The worms were paralysed and immobilized in a microfluidic device to allow controlled isoamyl alcohol (IAA) delivery [10]. After a 6 min pre-stimulus period, we presented six consecutive 30 s–on, 30 s–off IAA stimulations to the worm's nose. (i) Trigger-averaged AVA activity (±s.e.m.) in response to either IAA-offset (red) or control buffer-offset (black) at *t* = 10 s. Control buffer switches were performed in a separate set of animals to ensure that any activity responses are not due to pressure changes in the laminar flow device. (ii and iii) Single trials (*n* = 36 shifts across six animals for each condition). Statistically significant differences for the period shown by the green bars were calculated by a resampling procedure (Material and methods).

On average, AVA activity rises in response to IAA-off but not control buffer-off. However, panels (ii) and (iii) show that only about half of all animals responded. Most importantly, note the different pre-stimulus activity levels across trials within each condition: AVA responses are observed only for trials in which AVA activity was low at the time of stimulation. As discussed below, this result is crucial for understanding AVA sensory responses. Recording AVA activity for 6 min prior to the first stimulus allowed us to capture any spontaneous activity changes and measure the neuron's activity state at the time of stimulation, which clearly differed across trials. This difference is hidden if *F*_0_ is calculated from only the 10 s pre-stimulus period or only the stimulus window, rather than from the entire recording including the 6 min pre-stimulus period. Here, we calculate *F*_0_ from the neuron's natural Low state (Material and methods). Because many interneurons show spontaneous activity changes (see the main text), such long pre-stimulus recording periods are essential to (i) measure and report any spontaneous activity changes in the absence of acute sensory stimulation and (ii) determine the neuron's activity state at the time of stimulation.

(*b*) Step 2: Separate trials depending on neuron activity state at the time of stimulation and compare with control non-stimulated trials in the same state.

The same analysis as in (*a*) with trials separated depending on whether the stimulus arrived when AVA was in a High activity state (i) or in a Low activity state (ii). Kato *et al.* [20] and Gordus *et al*. [58] showed that AVA activity is bi-stable (see Material and methods for state classification). Colour bar in (*a*) applies to these panels.

This analysis revealed that if AVA is in a High activity state at the time of IAA-off, no response is observed (i). This is probably due to AVA's bi-stability: if AVA is already in a High state, it cannot rise further upon IAA-off. By contrast, if AVA is in a Low activity state pre-stimulus (ii), activity rises upon IAA-off much more compared with control buffer-off, because rises are observed in a higher fraction of animals.

This analysis is crucial, because AVA Low controls also show, on average, a spontaneous rise on the same timescale as do stimulated animals. We confirmed that AVA rises upon control buffer-off were indeed spontaneous, i.e. not induced by pressure change upon buffer switch, as the same change is observed during the pre-stimulus periods lacking buffer switches (data not shown). Furthermore, we can conclude that AVA response is, in fact, not very variable once pre-stimulus state is taken into account (80% responders in *b*(ii), versus 50% responders in *a*). These data lead to a new interpretation of IAA-off responses: High-state AVA does not appear to respond to this stimulus, while Low-state AVA responds reliably.

(*c*) Step 3: Convert activity changes to state probabilities to reveal how the stimulus affects behavioural state.

Many interneurons have been characterized to belong to either a forward or reverse command state ensemble (see the main text). For these neurons, the instantaneous brain-wide motor command state can be read out from the neuron's activity in a binary manner: for AVA, Rise and High correspond to reverse command state and Fall and Low correspond to forward command state [20]. For the AVA Low pre-stimulus trials, we binarized AVA activity according to these command states (Material and methods) and plotted the instantaneous probability of Rise + High states across trials (i). Panels (ii) and (iii) show rasters, with black corresponding to reversal command (Rise or High) state.

From this figure, we can read out that 80% of animals in a forward state respond to this stimulus by entering the reversal command state, whereas the spontaneous reversal command state probability seen in control recordings is only 40%. Importantly, this analysis highlights that the increased response amplitude in IAA-off in (*b*) can be accounted for by an increased response probability across trials rather than an increased response amplitude within each trial. These figures can be compared directly with recordings in freely moving animals to determine whether the immobilized preparation shows a successful sensorimotor transformation that can be further explored [20].

For an interneuron showing spontaneous activity changes that have not been as thoroughly characterized as AVAs, recordings made in freely moving animals can be used to link spontaneous activity to behaviour. An alternative is to record activity in immobilized animals while co-recording a well-characterized neuron like AVA to determine participation in shared motor command states.

Once these analyses have been performed, a further step is to assess whether sensory input impacts the behaviour-related activity of the neuron beyond biasing transition probabilities. If recordings are performed in immobilized animals, ideally several neurons are co-recorded, so that changes in correlations between interneurons can be assessed. In freely moving animals, it is important to quantify behaviour carefully to determine the full scope of interneuron activity correlations to behaviour. For example, AVA and AVE neurons show subtle but significant modulations in their activity slope or peak, respectively, depending on reversal speed [20]. Any stimulus that causes animals to perform slower reversals, such as O_2_ downshift, will lead to smaller AVA slopes and AVE peaks, results that might be misinterpreted as changes in these neurons' activities independent from their behaviour-related activities. On the other hand, careful quantifications might reveal a change in the relationship between interneuron activity and behaviour specifically due to sensory input, which might hint at a mechanism for sensorimotor integration (see the main text).

## 2. An updated view of sensorimotor control in *C. elegans*

Based on recent observations, a new big picture is emerging for how the worm brain could perform computations and generate decisions based on the sensory inputs it receives.

In previous work, many researchers have made the reasonably assumption that information flow from sensory to motor circuits is largely segregated and feed-forward. In such a model, changes in the environment are detected by sensory neurons and then transmitted to first- and second-layer interneurons, which potentially perform specialized computations on their inputs. For example, the sensory neuron AWA detects the odour diacetyl and signals to the first-layer interneuron AIA. While AWA is sensitive to diacetyl concentration and stimulus history, AIA filters this information and reports only the qualitative change (increase) in odour concentration [12]. In a different study, the first-layer interneuron AIY was shown to invert the sensory polarity of the odour-OFF neuron AWC into an odour-ON response in AIY. In contrast to AIY, first-layer interneuron AIB computes a temporally prolonged OFF response from AWC's input [10]. Both studies were performed in immobilized worms. The most intuitive conclusion from these studies was that dedicated first-layer interneurons perform computations on each sensory input; these interneurons then output transformed sensory information to premotor interneurons to ultimately control the animals' reorientation rate. Reversals are a major component of these reorientation manoeuvres; hence, the premotor interneuron AVA is the most prominent candidate to integrate the output of first- and second-layer interneuron computations. The hub neuron AVA is a bottleneck in the connectome, forming most of the chemical and electrical synapses onto A-class motor neurons [1,27], which execute the movements for reversals. Consistent with a premotor interneuron role, several studies showed that AVA activity in moving animals is tightly positively coupled to the reversal motor state [4,20,23–26]. Taken together, these studies suggested a feed-forward organization, with segregated circuits for stepwise sensory information processing (first- and second-layer interneurons) and motor command generation (premotor interneurons and motor neurons).

The first challenge to this interpretation was posed by calcium imaging experiments of primary sensory interneurons in freely crawling worms: surprisingly, all first- and second-layer interneurons mentioned above are strongly modulated by motor state. In freely moving animals, AIY shows activity changes in the absence of acute odorant stimulations. Here, AIY activity is correlated with the speed of forward locomotion and remains low or suppressed during reversal episodes [18]. These relationships between AIY activity and behaviour were observed as well in the presence of sensory input, when animals freely navigated salt gradients [19]. Also, AIA was found to be negatively modulated by reversal state; this modulation was preserved in the presence of a strong oxygen sensory stimulus [26]. In contrast to AIY and AIA, AIB interneurons are strongly positively coupled to the reversal state [19,20,26]; again, this activity–behaviour relationship is preserved in salt gradients [19] and under different oxygen conditions [26]. In addition, low amplitude calcium transients of AIB activity coincide with transient slowing manoeuvres during forward locomotion [20]; a fraction of these events then transit into reversals, which is when the highest amplitude signals in AIB can be observed [20]. Strikingly, high-amplitude calcium transients in AIB were coupled to reversals as reliably as activity of the premotor interneuron AVA; by this criterion, the classical primary sensory first-layer interneuron AIB would be indistinguishable from a typical premotor interneuron [20].

The second line of evidence suggesting a distributed rather than segregated representation of motor behaviours in *C. elegans* came from brain-wide calcium imaging experiments. In these experiments, worms are immobilized by muscle paralysis and confined to microfluidic channels; in the absence of both movement and experimentally induced acute sensory stimuli, surprisingly, about one-fourth of all measured head ganglia neurons are engaged in vigorous activity [20,28,29,37]. Currently, we assume that these activities are spontaneous, i.e. not triggered by transient sensory inputs. However, it is possible that dynamics are driven by tonic sensory neuron activity. For example, the confinement condition itself could represent a constant arousing stimulus. One has to keep further in mind that it might be practically impossible to perform such experiments under perfectly isotropic and constant conditions.

**Figure 2.**
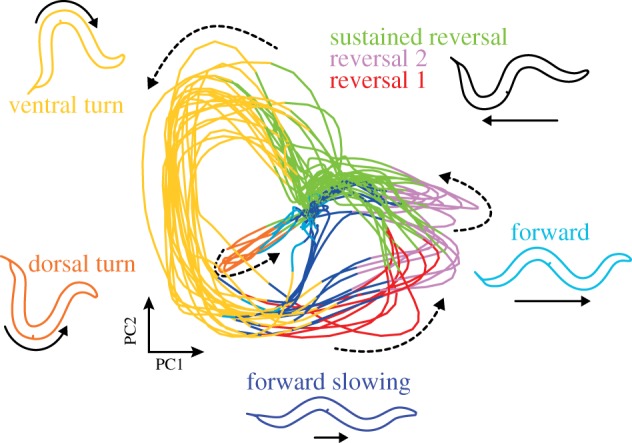
Phase plot of the first two principal components (PCs) of an 18 min long *C. elegans* brain-wide calcium imaging dataset from Kato *et al.* [20]. Activity of a subset of neurons with known motor output allows classification of brain state into different motor command states: forward, forward slowing, three reversal states, as well as ventral and dorsal turns (see [20]). These brain states are colour-coded, and schematics of the movements are shown. The brain state is continuously changing; the direction of brain state time evolution is shown by dashed arrows. Note that the manifold assembles individual behavioural commands into the major action sequence of the worm; moreover, discrete transitions between behavioural command states are embedded in a smooth progression of brain state.

Strikingly, most of these presumably spontaneous network activities were, however, highly structured across the neuronal population. Many interneurons, a set of head motor neurons, and body motor neurons in the retrovesicular ganglion exhibit coordinated collective network activity: AVB and RIB interneurons, SIA/B and RME head motor neurons and B-class motor neurons form a synchronized ensemble that activates in an antagonistic fashion to another ensemble of AVA, AVE, RIM and AIB interneurons along with A-class motor neurons. Transitions between these ensemble activities coincide with transient activations of RIV, SMDV and SMDD^2^ head motor neurons. Similar brain-wide correlations involving the aforementioned neuronal classes could be confirmed by pan neuronal calcium imaging in freely crawling animals [30,31], demonstrating that correlated brain-wide dynamics are not specific to the immobilized preparation. However, we described some qualitative differences in neuronal activity comparing freely moving with paralyzed worms [20], which will be discussed further below. The activity of most of these neurons can be subdivided into four discrete states, through which they cycle: Low, Rise, High and Fall [20]. Moreover, some neurons, primarily those of the AVB ensemble, exhibit fluctuating calcium levels during their high states. Representative neurons participating in brain dynamics were imaged in freely moving animals systematically; these data showed that these neurons encode behavioural states with a near-perfect reliability, comparable to the classical premotor interneurons: AVB ensemble High-state fluctuations correspond to changes in crawling speed during forward locomotion, while during reverse movement, these neurons are in the Low state and AVA ensemble neurons are in Rise or High states [20]. Computational analysis of brain-wide activity recordings showed that brain activity is bound by a low-dimensional attractor manifold, which can be visualized by principal components analysis (PCA) (figure 2). PCA reveals a neural state trajectory that recurrently revisits the same sub-regions, which correspond to particular neuronal ensemble activity patterns that are repeated in a cyclical fashion over time in a single recording (figure 2). The aforementioned calcium imaging recordings in freely moving worms enabled the assignment of a motor command to each sub-region, such that the cyclical progression of ensemble activities determines the cycle Forward crawling—Forward Slowing—Reverse crawling—Dorsal or Ventral turn—Forward crawling. Hence, the attractor manifold is a neuronal representation of the worm's major action cycle [20] (figure 2).

In summary, the motor command activities of the classical premotor interneurons are only a small part of globally distributed network states that also reliably recruit first- and second-layer interneurons such as AIB and RIB, along with motor neuron outputs. This finding calls for a major reappraisal of earlier results. However, first, we have to consider some caveats. First, there are cases where modulation of calcium transients does not follow modulation of membrane potential [32,33]. Second, the calcium transients in AIY, AIA and AIZ are typically measured in neurites [10–12,15,18], and hence could not be detected in brain-wide recordings of nuclear-localized calcium indicators (though, note that one study reported nuclear calcium signals in AIY [34]). It therefore remains to be shown whether AIY, AIA and AIZ are additional members of the motor command ensembles. Alternatively, behaviour-correlated activities in these neurons could result from proprioceptive feedback as a direct consequence of movement and thus be non-observable in immobilized worms. This question should be addressed in any future experiment examining these neurons side by side in immobilized and freely moving animals (box 2).

Could behaviour-related signals in first- and second-layer interneurons in immobilized or moving animals be a consequence of pure feedback (i.e. efference copy) from premotor interneurons or others driving the motor state? Two different results suggest that feedback cannot be the only purpose of this activity. First, when premotor interneuron AVA was inhibited via a histamine-gated chloride channel, preventing animals from executing reversals [20,35], the attractor manifold was remarkably robust to this manipulation: other neurons of the reversal ensemble still generated the reversal command in a manner almost indistinguishable from unperturbed animals, and only the transmission of this command information to motor neurons was affected. As a result, freely moving worms interrupt the action cycle during reverse command states and they freeze instead of reversing; however, the brain still attempts to execute the reversal [20]. A second piece of evidence suggesting that first- and second-layer interneuron behaviour-related activities act as command signals is the robustness with which their manipulation affects behaviour. For example, AIB and AIZ ablation greatly reduces reversal frequency [9,18] while activation of these neurons triggers reversals [17,18,36]. The opposite effects have been found when activating AIY and RIB [18]. Altogether, these data suggest that motor commands are generated in a distributed, brain-wide manner that includes first-layer classical ‘primary sensory’ interneurons (figure 1*b*).

The global brain dynamics representing motor commands were surprisingly robust to sensory inputs. Strong oxygen stimuli, switches between 4 and 21% oxygen, did not alter the relationship between interneuron activity and global motor command states. Hence, the shape of the attractor manifold was largely unaffected. However, an effective sensory to motor transformation was observed, as the stimuli entrained the instantaneous phase on the action command cycle: the reverse motor command state occurred with increased or decreased probabilities at high versus low oxygen, respectively [20,37]. These observations were surprising, because oxygen sensory neurons directly synapse onto AVA, AVE and RIB, which still show strong motor command signals but lacked purely sensory evoked activity patterns; these neurons, in fact, together receive a large portion of synaptic output from O_2_ sensory neurons. Moreover, only a few sensory-tuned interneurons were observed in these recordings: RIG reported in [20] and AUA and RMG reported in [37]. Calcium imaging of AVA, AVE and RIB in freely moving animals exposed to oxygen shifts confirmed that these neuronal classes retain their strict tuning to motor state under stimulus conditions [20] (box 1). These results showed that the attractor manifold is largely robust to sensory stimulation. Similar preserved behaviour-correlated interneuron activities were observed in freely moving animals, during salt gradient navigation [19] and in other neurons in different oxygen environments [26], despite effective sensory-induced changes in behaviour in both studies. In other words, sensory inputs trigger, in a probabilistic manner, pre-patterned network activity that is nearly indistinguishable from spontaneous activity.

It remains to be shown whether these features hold when worms are presented with more complex multisensory inputs, or engaged in behaviours other than the aforementioned action cycle such as mating [38] or nictation [39]. Moreover, it is still unclear to what extent the manifold can encode or control the postural diversity in worm behaviour that has been reported [40], or three-dimensional locomotion [41]. So far, studies have shown that the manifold is preserved under different developmental [37] and feeding [29] conditions. Still, analyses of larger neuronal populations in mammals have shown that neuronal dimensionality can vary with stimulus complexity [42], task complexity [43] and recording time [43]. In line with these considerations, we have observed much more variation in our recordings of neural activity in freely moving compared to paralyzed worms ([20], figure S3; our observations); these variations often correspond to variations in behaviour. We therefore expect the precise structure of the manifold to depend on recording conditions, and expect that the manifold described in paralyzed, unstimulated animals in [20] will be seen as a simple scaffold as more complex recording conditions are used.

Motor state modulation of neuronal activity must not be exclusive to interneurons but perhaps extends to a subset of sensory neuron classes. Decreases in ambient oxygen levels evoked highly variable calcium fluctuations in BAG sensory neurons [22]. Kato *et al*. [20] showed that these fluctuations are correlated with brain state, i.e. BAG sensory responses are transiently attenuated during reversal command states. Ghosh *et al*. [44] showed that feedback from RIM affects ASH sensory responses to ultimately affect decision-making. Moreover, Chalasani *et al*. [11] showed that neuromodulatory feedback from AIA to AWC shapes the dynamics of AWC sensory responses and subsequent behaviours. These results are important hints that motor state modulation and feedback mechanisms in *C. elegans* potentially extend to the domain of primary sensory neurons.

While several studies together have shown that sensory stimulation does not produce qualitative changes in interneuron relationships to motor command state [19,20,26], subtle effects were reported [20,26], the consequences of which remain to be explored. Such small quantitative alterations could be signatures of or even underlie observed changes in behavioural state transition probability or other parameters of locomotion, such as crawling speed. One case in which subtle modulations of ongoing behaviour-related dynamics have been shown to implement a sensorimotor transformation is in the interneuron RIA. Two seminal studies showed that the RIA neurite has three compartments with independent calcium dynamics, that two of these compartments receive input from motor neurons regarding the worm's head-bend direction while the third receives sensory input, and that sensory input modulates the motor-related dynamics in RIA to ultimately bias the worm's head-bend [45,46]. This sensorimotor programme allows the worm to steer towards an odour source [46]. Whether such compartmentalized calcium dynamics are common in the worm remains unknown. Whether or not compartmentalization is widespread, it remains unclear how such activity modulations might act on the population of recurrently connected interneurons that underlies forward/reverse locomotion switches. See box 2 for a description of how a typical interneuron sensory response experiment could be analysed to begin to address this question. We propose hypotheses in the following section that take into account the functional implications of distributed, mixed sensorimotor representations.

## 3. Putting behaviour first: towards a new understanding of sensorimotor circuitry

These recent findings suggest that we must reimagine how to best perform and interpret experiments involving sensorimotor transformations. With nearly all interneurons showing spontaneous motor command dynamics, researchers must first describe and understand those ongoing dynamics under their recording conditions before studying sensory input responses (box 1 and box 2). In light of this, in this section, we will first focus on how the brain generates behaviour in the absence of experimentally evoked sensory stimulation. Only after exploring these ideas does it make sense to hypothesize how sensory stimuli are incorporated into ongoing behaviour-related dynamics. In our view, the worm brain is best understood as a distributed and dynamic system meant to produce appropriate behaviour, which involves most interneurons and motor neurons. This distributed network receives inputs from sensory neurons that act to modify its dynamics for the sake of decision-making.

First, how do we best understand the ongoing production of behaviour? Motor commands are best summarized by the attractor manifold, a low-dimensional representation of high-dimensional neuronal activity. There are three major features of network activity in the worm that contribute to the manifold: (i) the participation of a relatively large number of neurons, (ii) strong correlations and anti-correlations between them and (iii) a cyclical repetition of network activity states that reproducibly recruit the same classes of neurons in each cycle [20]. What could be the functional advantage of such a low-dimensional dynamical system coding for behaviour?

The participation of many neurons in motor command generation raises the possibility that information about behaviour is encoded at the level of the population; if this is the case, behaviour cannot be fully decoded from individual neuron traces alone. Population codes were shown in larger neuronal networks of various model organisms ranging from invertebrates to rodents and primates [47–52]. However, some key differences have to be noted: for example, in large spiking cortical networks, neurons with similar anatomical or functional features can be represented in great quantities, but in single trials the participation of individual neurons is often sparse and variable; both have substantial impact on the properties of the underlying population code [53]. Even in larger invertebrates, for example in an *Aplysia* motor ganglion, rhythmic motor patterns arise as an emergent property of neuronal populations [50]. Unlike in cortex, in the compacted worm brain, most neuronal classes are represented only by two bilaterally symmetric members and their recruitment to the neuronal ensemble is very reliable in single trials. As a result, the motor states of the action cycle can be easily inferred from the activity of few or even single neurons [20], suggesting that population coding for discrete motor state might not be the most salient feature of the system.

On the other hand, a prominent neuronal population-level feature found in *C. elegans* is the smoothness of the attractor manifold, i.e. the lack of abrupt switches and fixed points (figure 2). This smooth transitioning of network activity might serve the need to gradually adjust metrics of motion while animals switch between different locomotor programmes. For example, the transition from forward to backward crawling, which are two incompatible movement patterns, and which are generated by distinct and dedicated motor neuron circuits (B- versus A-class motor neurons), is accompanied by smooth deceleration and acceleration of locomotion speed [20,26] (see also Roberts *et al*. [5], who reported an intermittent discrete pause state). Imagine, you are driving your car through the city to find a parking spot; before you can back up and turn into a free spot, you need to first hit the breaks to slow your vehicle down; hence your discrete action of switching to the reverse gear must be embedded in another smoothly executed action command. To test this population coding hypothesis, it will be necessary to verify in a quantitative manner that population-level features are not trivial or epiphenomenal outcomes of simple primary features found already in individual neurons; sophisticated computational methods for scrutinizing these hypotheses have been recently developed [54]. Moreover, whole-brain imaging in freely moving worms should reveal whether other possible population-level features have indeed behavioural correlates.

Whether or not population coding exists in worms, strongly correlated and reliably recruited network activity underlies motor commands in the worm. We propose that one purpose of these features is the ability to robustly produce behaviour: in this view, the attractor manifold provides a backbone of pre-patterned network activity that persists under a stream of complex multisensory inputs, like the ones worms must encounter in their natural soil habitats. In this way, understanding the motor command system is a prerequisite for understanding how sensory inputs are processed. A striking feature of the interneurons that are recruited to motor command ensembles is their privileged role in the *C. elegans* connectome: due to their exceptional high in- and outdegree connectivity [27] and interconnectivity among themselves, they form a so-called rich club [55]. They receive plenty of synaptic input from primary sensory interneurons as well as directly from primary sensory neurons. This provides ample potential regulatory knobs by which sensory inputs can control behaviour. We hypothesize that transition probabilities on the attractor manifold depend both on internal dynamics and on a weighted function of sensory-related inputs received by interneurons. For example, when the system is in the forward command state, the probability of transiting to the reversal command state can be pushed towards high values by strong nociceptive inputs to acutely trigger escape reversals; other subtle inputs and internal dynamics could modulate the basal, perhaps stochastic, rate of ‘spontaneous’ transitions. Both the synaptic strengths and the identity of the receiving interneuron could be a means to modulate the weights function. This also explains the apparent redundancy in the motor command-related activity patterns of different interneurons: in this view, individual interneurons are not redundant but represent channels that can differentiate inputs into a distributed motor command network, while participating in that network by receiving and sending signals that affect ongoing dynamics.

Importantly, the behaviours encoded by brain-wide dynamics are high level. The cyclical progression of brain state along the attractor manifold, like the underlying activity patterns of individual neurons, should not be mistaken for a central-pattern-generator circuit for the undulatory movement of the worm; i.e. while it is possible to decode the worm's progression through the action command cycle, there is little or no information about its exact instantaneous posture dynamics like the dorsoventral bending pattern. Note again that neurons like RIB encode the speed of locomotion but not the underlying oscillatory movement. Hence, the action command cycle in the brain conveys high-level control variables of behaviour. This control model is consistent with a wide variety of results in the literature. Optogenetics experiments targeting individual neuron classes can modulate the probability of motor state transitions [17,18,36], and closed-loop optogenetic control of just the AIY neurons was sufficient to achieve steering in a virtual gradient landscape [36]. Furthermore, in [9], ablation of individual interneurons resulted in differential effects on behavioural state; the authors consequently proposed a similar control model for the role of individual interneurons (without accounting for their additional role in motor command generation) [9]. In line with this role in high-level control, these studies reported no obvious effects of interneuron ablations and activations on posture dynamics, but rather showed that these perturbations biased the probabilities of behaviours to occur more or less often, but with normal coordination (except in cases where motor neurons are also affected [4]). Still, finer-grained measurements may be required to quantify possible effects of neuronal ablations on posture dynamics; for examples, see [56,57]. These experiments demonstrate that inputs via individual channels can, in principle, be sufficient to trigger behaviours, and in the context of our interpretation, the distributed network must be sensitive not only to motor command dynamics but also to individual sensory input channels. This view expands on the results of Gordus *et al*. [58] by suggesting that the integration of motor commands and sensory inputs takes place not only in primary sensory interneuron AIB but also in a vast majority of interneurons, including premotor interneurons [20]. In future work, multisensory stimulation experiments and optogenetics combined with whole-brain imaging can test this hypothesis. Moreover, determining properties of the proposed weights function (e.g. linear versus nonlinear processes) will be crucial to understanding the computations and decision-making processes of this system. Interestingly, in mice, it was recently shown that sensory inputs do not generate new behaviours but instead bias a set of spontaneous behaviours [59], just like sensory inputs bias spontaneous reversal probability in worms. Hence, these principles found in *C. elegans* might apply and scale to the larger brains of rodents. Our prediction is that the multitude of stereotypic motor motifs of mice might be encoded by and can be selected from pre-patterned, perhaps more plastic and higher dimensional, attractor dynamics in motor areas of the rodent brain.

We argue here that the studies describing sensory computations performed by interneurons AIA, AIY and AIB [10,12], mentioned in the previous section, do not necessarily contradict the imaging studies performed in freely moving worms. However, the exciting and important challenge for future studies is to address how and why these neurons multiplex. One possibility is that both functions are compartmentalized at the subcellular or even molecular level. Alternatively, these neurons exhibit mixed tuning, i.e. their activity is instantaneously computed from both motor and sensory inputs.

Mixed neuronal representations in individual neurons is not a peculiarity of the compacted nematode nervous system. In both rodent and primate cortex, the same neurons can be tuned to several task parameters, like sensory inputs, decision variables and behaviour [60]. It has been proposed that mixed representations can enhance the coding and decoding ability of neuronal activity in these larger neuronal networks (see [61] for details). Whether such principles can be found in a downscaled form in *C. elegans* remains to be shown. A prerequisite to address these questions is to record activity of interneuron populations in freely moving worms performing tasks in response to sensory stimuli, ideally in multisensory environments; then, the relative contributions of motor variables and sensory inputs to neuronal activity must be carefully disentangled.

At the mechanistic level, RIA neurons solve the problem of multiplexing signals within one cell by compartmentalizing them in different subcellular domains of the neurite. So far, no study reported compartmentalized calcium domains in the processes of other primary sensory interneurons AIY, AIZ and AIA; hence, we must assume that sensory and motor information to be truly mixed in these neurons. However, it should be noted that neuronal compartmentalization could be more common than currently accounted for; for example, electron microscopy studies show that many neurons have spatially segregated synaptic domains which could indicate compartmentalization [1]. Calcium imaging in animals freely navigating odorant gradients should give new insights into the function of these mixed representations. One possibility that we propose is that sensory processing in interneurons is gated by motor state: for example, AIY might only process sensory inputs while the worm is moving forward and ignores them during reversals. Because AIY activity is modulated by forward crawling speed, this gating mechanism could be a means of active gain control. In this model, animals would be more sensitized to certain inputs when in a fast crawling mode, e.g. during roaming periods [62,63] or in the absence of food [5]. In this line, previous work showed that behavioural responses to thermal stimuli depended on the locomotory phase of the worm's posture [64]. Moreover, higher-level behavioural context gating of sensory response was recently reported [65,66]. It therefore will be necessary to further develop behavioural paradigms where neural circuit activity can be studied under controllable open- and closed-loop conditions, i.e. like in Kocabas *et al*. [36]. Interestingly, movement modulates neuronal activity in sensory cortical areas in the mouse [67,68]. In *Drosophila*, both walking and flying strongly modulate the activity of neurons early in the visual pathway [69,70]. This modulation persists in blinded flies and its strength is tuned quantitatively to movement parameters, similar to worm interneuron activities that show a quantitative representation of movement speed. Cohn *et al*. [71] showed that the fly's instantaneous behavioural state, represented by dopamine neuron ensemble activity, could drive plasticity of odour responses. These studies show that behavioural state can have a pronounced effect on higher sensory processing areas in more complex brains.

Other possible functions besides gain modulation could be to predict the effects of movements on sensation, as occurs during processing of visual flow fields in rodents and flies [72,73]. Moreover, theories of motor control suggest that efference copies of motor commands feed into internal forward models that predict the outcomes of movements, which then can be optimized based on detecting a prediction error [74,75]. In this context, we suggest that the widespread motor command representation in worms could be integrated with proprioceptive feedback to optimize the execution of behaviour. This hypothesis is supported by our observations that the reversal motor command is sustained in pharmacologically paralyzed as well as in physically constrained animals, compared to untreated unrestrained worms. This suggests that information about lack of movement is conveyed to the brain to prolong the motor command [20]. *C. elegans* could be a tractable system to scrutinize these hypotheses.

In fact, it appears that brain-wide representations of ongoing multi-dimensional behaviour also dominate mouse brain activity [76,77]. Behaviour representations explain the vast majority of variance in these brain-wide datasets, and they persist unperturbed by, and in orthogonal dimensions to, both sensory input [76] and multi-stage decision-making task parameters [77]. Both studies address similar issues of data interpretation as we describe in box 1, and Musall *et al.* [77] point out that many results in the rodent decision-making literature may require re-evaluation in light of these findings, as we have argued here for *C. elegans* literature. Multi-dimensional behaviour-related activity also pervades sensory cortices, leading [76] to argue that sensory cortex function will probably only be understood in relation to the animal's ongoing behaviour, as we argue for primary sensory interneuron function in worms. In conclusion, work across phyla suggests that concurrent, distributed integration of sensory and motor dynamics is a fundamental principle of nervous system function. Hence, we propose *C. elegans* as a tractable model to study the functions and computational advantages of these brain–body–environment interactions.

## 4. Material and methods

### (a) Calcium imaging in freely moving animals with O_2_ delivery

Data in box 1 are from Kato *et al.* [20]. Briefly, the automatic re-centering system described in [24] was used to keep the worm's head centred on the objective. Young adult worms expressing GCaMP and mCherry in AVA under the *flp-18* promoter were recorded on an inverted microscope, moving freely on an agar surface in an airtight chamber with inlet and outlet connectors for gas flow delivery. Simultaneous recordings of animal behaviour were made via infrared illumination and an additional low-magnification objective and camera from above. A GCaMP and mCherry ratio was calculated to correct for motion artefacts, and the mean of the bottom 10% of the data was used as *R*_0_ to calculate (*R*−*R*_0_)/*R*_0_ = Δ*R*/*R*_0_.

### (b) Calcium imaging in immobilized worms with isoamyl alcohol delivery

Animals were immobilized using 1 mM tetramisole and restricted in the microfluidic four-flow device for olfactory stimulation described in [10]. This device uses laminar flow to ensure little to no diffusion between the two buffers that make contact with the worm's nose. Recordings were 12 min long; in both stimulus and control recordings, for the first 6 min, NGM + fluorescein (20 mM) buffer contacted the worm's nose. Then, every 30 s, this buffer was switched with a buffer lacking fluorescein, either NGM + IAA (9.2 × 10^−4^ M) in the stimulus condition or NGM alone in the control condition. Fluorescence intensity measurements near the worm's nose were used to quantify fluorescein levels to ensure laminar flow and to determine stimulation times.

Nuclear-localized GCaMP5 K was expressed in AVA under the *flp-18* promoter (strain: ZIM747; *lite-1* (*xu7*); *mzmEx458* = [P*flp-18*::mCherry-His58; P*flp-18*::NLSGCaMP5 K]). Images were acquired on an inverted compound microscope (Zeiss Axio Observer.Z1) using a CCD camera (Evolve 512, Photometrics). Excitation light (470 nm) was provided by a CoolLED pE excitation system. A 40× oil immersion objective (N/A 1.3) was used to obtain unbinned images at 33 ms exposure time with the VisiView software (Visitron Systems GmbH, Germany).

### (c) Resampling test

The combined data from the last 20 s (horizontal bars in the figure of box 2) of the IAA-off or control buffer-off stimulations were randomly redistributed into two surrogate control groups. The number of trials in each surrogate sample was the same as in the two experimental conditions. In each of 1 million iterations, we calculated the difference between the integrals of the surrogate IAA sample and the surrogate control sample. The *p*-values are the fraction of results when the distance between the resampled datasets was equal or larger than the observed one; hence, they estimate the probability that the observed differences occurred by chance.

### (d) Neuron states

Many *C. elegans* interneurons are bi-stable, displaying mainly Low and High states, as well as falls and rises when transition between the states. We performed phase-segmentation on the AVA traces into Low, Rise, High and Fall as was described in [20]. Briefly, periods were classified as rises when the time derivative of the trace was higher than a small and manually determined threshold, while periods were classified as falls if the time derivative was lower than another threshold. High and Low states were then assigned to remaining periods based on lawful order and a threshold. In box 2, High state corresponds to combined Rise and High, which for AVA is known to correspond to reversal behaviour [20], while Low corresponds to combined Fall and Low, known to correspond to forward command state. Classification into High and Low in box 2 required these states to be constant for 10 s pre-stimulus.

## Endnotes

1 The terms ‘command interneurons’ and ‘premotor interneurons’ have been used interchangeably in the literature; here we use ‘premotor interneurons’ as recent work suggests more distributed command generation, as discussed below.

2 In the original work [18,28], the identity of these neurons was ambiguous between SMBD, SMBV and SMDD. We now confirm that across all recordings in [20], these identities belong to the SMDD class.

## Data accessibility

This article has no additional data.

## References

[1] White JG, Southgate E, Thomson JN, Brenner S 1986 The structure of the nervous system of the nematode *Caenorhabditis elegans*. Philos. Trans. R. Soc. Lond. B 314, 1–340. (10.1098/rstb.1986.0056) 22462104

[2] Emmons SW 2015 The beginning of connectomics: a commentary on White *et al*. (1986) ‘The structure of the nervous system of the nematode *Caenorhabditis elegans*'. Philos. Trans. R. Soc. B 370, 20140309 (10.1098/rstb.2014.0309) PMC4360118 25750233

[3] Chalfie M, Sulston JE, White JG, Southgate E, Thomson JN, Brenner S 1985 The neural circuit for touch sensitivity in *Caenorhabditis elegans*. J. Neurosci. 5, 956–964. (10.1523/JNEUROSCI.05-04-00956.1985) 3981252 PMC6565008

[4] Kawano T, Po MD, Gao S, Leung G, Ryu WS, Zhen M 2011 An imbalancing act: gap junctions reduce the backward motor circuit activity to bias *C. elegans* for forward locomotion. Neuron. 72, 572–586. (10.1016/j.neuron.2011.09.005) 22099460

[5] Roberts WM et al. 2016 A stochastic neuronal model predicts random search behaviors at multiple spatial scales in *C. elegans*. eLife 5, 489 (10.7554/eLife.12572) PMC4798983 26824391

[6] Tsalik EL, Hobert O 2003 Functional mapping of neurons that control locomotory behavior in *Caenorhabditis elegans*. J. Neurobiol. 56, 178–197. (10.1002/neu.10245) 12838583

[7] Gabel CV, Gabel H, Pavlichin D, Kao A, Clark DA, Samuel ADT 2007 Neural circuits mediate electrosensory behavior in *Caenorhabditis elegans*. J. Neurosci. 27, 7586–7596. (10.1523/JNEUROSCI.0775-07.2007) 17626220 PMC6672606

[8] Iino Y, Yoshida K 2009 Parallel use of two behavioral mechanisms for chemotaxis in *Caenorhabditis elegans*. J. Neurosci. 29, 5370–5380. (10.1523/JNEUROSCI.3633-08.2009) 19403805 PMC6665864

[9] Gray JM, Hill JJ, Bargmann CI 2005 A circuit for navigation in *Caenorhabditis elegans*. Proc. Natl Acad. Sci. USA 102, 3184–3191. (10.1073/pnas.0409009101) 15689400 PMC546636

[10] Chalasani SH, Chronis N, Tsunozaki M, Gray JM, Ramot D, Goodman MB, Bargmann CI 2007 Dissecting a circuit for olfactory behaviour in *Caenorhabditis elegans*. Nature 450, 63-70. (10.1038/nature06292) 17972877

[11] Chalasani SH, Kato S, Albrecht DR, Nakagawa T, Abbott LF, Bargmann CI 2010 Neuropeptide feedback modifies odor-evoked dynamics in *Caenorhabditis elegans* olfactory neurons. Nat. Neurosci. 13, 615–621. (10.1038/nn.2526) 20364145 PMC2937567

[12] Larsch J, Flavell SW, Liu Q, Gordus A, Albrecht DR, Bargmann CI 2015 A circuit for gradient climbing in *C. elegans* chemotaxis. Cell Rep. 12, 1748–1760. (10.1016/j.celrep.2015.08.032) 26365196 PMC5045890

[13] Oda S, Tomioka M, Iino Y 2011 Neuronal plasticity regulated by the insulin-like signaling pathway underlies salt chemotaxis learning in *Caenorhabditis elegans*. J. Neurophysiol. 106, 301–308. (10.1152/jn.01029.2010) 21525368

[14] Kunitomo H, Sato H, Iwata R, Satoh Y, Ohno H, Yamada K, Iino Y 2013 Concentration memory-dependent synaptic plasticity of a taste circuit regulates salt concentration chemotaxis in *Caenorhabditis elegans*. Nat. Commun. 4, 1–11. (10.1038/ncomms3210) 23887678

[15] Clark DA, Biron D, Sengupta P, Samuel ADT 2006 The AFD sensory neurons encode multiple functions underlying thermotactic behavior in *Caenorhabditis elegans*. J. Neurosci. 26, 7444–7451. (10.1523/JNEUROSCI.1137-06.2006) 16837592 PMC6674189

[16] Guillermin ML, Carrillo MA, Hallem EA 2017 A single set of interneurons drives opposite behaviors in *C. elegans*. Curr. Biol. 27, 2630–2639.e6 (10.1016/j.cub.2017.07.023) 28823678 PMC6193758

[17] Piggott BJ, Liu J, Feng Z, Wescott SA, Xu XZS 2011 The neural circuits and synaptic mechanisms underlying motor initiation in *C. elegans*. Cell 147, 922–933. (10.1016/j.cell.2011.08.053) 22078887 PMC3233480

[18] Li Z, Liu J, Zheng M, Xu XZS 2014 Encoding of both analog- and digital-like behavioral outputs by one *C. elegans* interneuron. Cell 159, 751–765. (10.1016/j.cell.2014.09.056) 25417153 PMC4243084

[19] Luo L et al. 2014 Dynamic encoding of perception, memory, and movement in a *C. elegans* chemotaxis circuit. Neuron 82, 1115–1128. (10.1016/j.neuron.2014.05.010) 24908490 PMC4082684

[20] Kato S, Kaplan HS, Schrödel T, Skora S, Lindsay TH, Yemini E, Lockery S, Zimmer M 2015 Global brain dynamics embed the motor command sequence of *Caenorhabditis elegans*. Cell 163, 1–50. (10.1016/j.cell.2015.09.034) 26478179

[21] Pierce-Shimomura JT, Morse TM, Lockery SR 1999 The fundamental role of pirouettes in *Caenorhabditis elegans* chemotaxis. J. Neurosci. 19, 9557–9569. (10.1523/JNEUROSCI.19-21-09557.1999) 10531458 PMC6782915

[22] Zimmer M et al. 2009 Neurons detect increases and decreases in oxygen levels using distinct guanylate cyclases. Neuron 61, 865–879. (10.1016/j.neuron.2009.02.013) 19323996 PMC2760494

[23] Arous JB, Tanizawa Y, Rabinowitch I, Chatenay D, Schafer WR 2010 Automated imaging of neuronal activity in freely behaving *Caenorhabditis elegans*. J. Neurosci. Methods 187, 229–234. (10.1016/j.jneumeth.2010.01.011) 20096306

[24] Faumont S et al. 2011 An image-free opto-mechanical system for creating virtual environments and imaging neuronal activity in freely moving *Caenorhabditis elegans*. PLoS ONE 6, e24666 (10.1371/journal.pone.0024666) 21969859 PMC3182168

[25] Larsch J, Ventimiglia D, Bargmann CI, Albrecht DR 2013 High-throughput imaging of neuronal activity in *Caenorhabditis elegans*. Proc. Natl Acad. Sci. USA 110, E4266–E4273. (10.1073/pnas.1318325110) 24145415 PMC3831453

[26] Laurent P, Soltesz Z, Nelson GM, Chen C, Arellano-Carbajal F, Levy E, De Bono M 2015 Decoding a neural circuit controlling global animal state in *C. elegans*. eLife 4, e4241 (10.7554/eLife.04241) PMC4440410 25760081

[27] Varshney LR, Chen BL, Paniagua E, Hall DH, Chklovskii DB 2011 Structural properties of the *Caenorhabditis elegans* neuronal network. PLoS Comput. Biol. 7, e1001066 (10.1371/journal.pcbi.1001066) 21304930 PMC3033362

[28] Schrödel T, Prevedel R, Aumayr K, Zimmer M, Vaziri A 2013 Brain-wide 3D imaging of neuronal activity in *Caenorhabditis elegans* with sculpted light. Nat. Methods. 10, 1013–1020. (10.1038/nmeth.2637) 24013820

[29] Skora S, Mende F, Zimmer M 2018 Energy scarcity promotes a brain-wide sleep state modulated by insulin signaling in *C. elegans*. Cell Rep. 22, 953–966. (10.1016/j.celrep.2017.12.091) 29386137 PMC5846868

[30] Nguyen JP, Shipley FB, Linder AN, Plummer GS, Liu M, Setru SU, Shaevitz JW, Leifer AM 2016 Whole-brain calcium imaging with cellular resolution in freely behaving *Caenorhabditis elegans*. Proc. Natl Acad. Sci. USA 113, E1074–E1081. (10.1073/pnas.1507110112) 26712014 PMC4776509

[31] Venkatachalam V et al. 2016 Pan-neuronal imaging in roaming *Caenorhabditis elegans*. Proc. Natl Acad. Sci. USA 113, E1082– E1088. (10.1073/pnas.1507109113) 26711989 PMC4776525

[32] Williams PDE, Zahratka JA, Rodenbeck M, Wanamaker J, Linzie H, Bamber BA 2018 Serotonin disinhibits a *Caenorhabditis elegans* sensory neuron by suppressing Ca^++^-dependent negative feedback. J. Neurosci. 1908, 17 (10.1038/nature24056) PMC5824741 29358363

[33] Zahratka JA, Williams PDE, Summers PJ, Komuniecki RW, Bamber BA 2015 Serotonin differentially modulates Ca^2+^ transients and depolarization in a *C. elegans* nociceptor. J. Neurophysiol. 113, 1041–1050. (10.1152/jn.00665.2014) 25411461 PMC4329441

[34] Kotera I, Tran NA, Fu D, Kim J, Rodgers JB, Ryu WS 2016 Pan-neuronal screening in *Caenorhabditis elegans* reveals asymmetric dynamics of AWC neurons is critical for thermal avoidance behavior. eLife 5, pii:e19021 (10.7554/eLife.19021) PMC5142811 27849153

[35] Pokala N, Liu Q, Gordus A, Bargmann CI 2014 Inducible and titratable silencing of *Caenorhabditis elegans* neurons in vivo with histamine-gated chloride channels. Proc. Natl Acad. Sci. USA 111, 2770–2775. (10.1073/pnas.1400615111) 24550306 PMC3932931

[36] Kocabas A, Shen C-H, Guo ZV, Ramanathan S 2012 Controlling interneuron activity in *Caenorhabditis elegans* to evoke chemotactic behaviour. Nature 490, 273–277. (10.1038/nature11431) 23000898 PMC4229948

[37] Nichols ALA, Eichler T, Latham R, Zimmer M 2017 A global brain state underlies *C. elegans* sleep behavior. Science 356, eaam6851 (10.1126/science.aam6851) 28642382

[38] Emmons SW 2018 Neural circuits of sexual behavior in *Caenorhabditis elegans*. Annu. Rev. Neurosci. 41, 1 (10.1146/annurev-neuro-070815-014056) 29709211

[39] Lee H, Choi M-K, Lee D, Kim H-S, Hwang H, Kim H, Park S, Paik Y, Lee J 2011 Nictation, a dispersal behavior of the nematode *Caenorhabditis elegans*, is regulated by IL2 neurons. Nat. Neurosci. 15, 107–112. (10.1038/nn.2975) 22081161

[40] Brown AEX, Yemini EI, Grundy LJ, Jucikas T, Schafer WR 2012 A dictionary of behavioral motifs reveals clusters of genes affecting *Caenorhabditis elegans* locomotion. Proc. Natl Acad. Sci. USA 110, 791–796. (10.1073/pnas.1211447110) 23267063 PMC3545781

[41] Bilbao A, Patel AK, Rahman M, Vanapalli SA, Blawzdziewicz J 2018 Roll maneuvers are essential for active reorientation of *Caenorhabditis elegans* in 3D media. Proc. Natl Acad. Sci. USA 115, E3616–E3625. (10.1073/pnas.1706754115) 29618610 PMC5910807

[42] Cowley BR, Smith MA, Kohn A, Yu BM 2016 Stimulus-driven population activity patterns in macaque primary visual cortex. PLoS Comput. Biol. 12, e1005185 (10.1371/journal.pcbi.1005185) 27935935 PMC5147778

[43] Gao P et al. 2017 A theory of multineuronal dimensionality, dynamics and measurement. bioRxiv. 214262 (10.1101/214262)

[44] Ghosh DD et al. 2016 Neural architecture of hunger-dependent multisensory decision making in *C. elegans*. Neuron 92, 1049–1062. (10.1016/j.neuron.2016.10.030) 27866800 PMC5147516

[45] Hendricks M, Ha H, Maffey N, Zhang Y 2012 Compartmentalized calcium dynamics in a *C. elegans* interneuron encode head movement. Nature 487, 99–103. (10.1038/nature11081) 22722842 PMC3393794

[46] Liu H, Yang W, Wu T, Duan F, Soucy E, Jin X, Zhang Y 2018 Cholinergic sensorimotor integration regulates olfactory steering. Neuron 97, 390–405. (10.1016/j.neuron.2017.12.003) 29290549 PMC5773357

[47] Mazor O, Laurent G 2005 Transient dynamics versus fixed points in odor representations by locust antennal lobe projection neurons. Neuron 48, 661–673. (10.1016/j.neuron.2005.09.032) 16301181

[48] Parnas M, Lin AC, Huetteroth W, Miesenböck G 2013 Odor discrimination in *Drosophila*: from neural population codes to behavior. Neuron 79, 932–944. (10.1016/j.neuron.2013.08.006) 24012006 PMC3765961

[49] Briggman KL, Abarbanel HDI, Kristan WB 2005 Optical imaging of neuronal populations during decision-making. Science 307, 896–901. (10.1126/science.1103736) 15705844

[50] Bruno AM, Frost WN, Humphries MD 2017 A spiral attractor network drives rhythmic locomotion. eLife 6, 471 (10.7554/eLife.27342) PMC5546814 28780929

[51] Mante V, Sussillo D, Shenoy KV, Newsome WT 2013 Context-dependent computation by recurrent dynamics in prefrontal cortex. Nature 503, 78–84. (10.1038/nature12742) 24201281 PMC4121670

[52] Churchland MM, Cunningham JP, Kaufman MT, Foster JD, Nuyujukian P, Ryu SI, Shenoy KV 2012 Neural population dynamics during reaching. Nature 487, 51–56. (10.1038/nature11129) 22722855 PMC3393826

[53] Schneidman E 2016 Towards the design principles of neural population codes. Curr. Opin. Neurobiol. 37, 133–140. (10.1016/j.conb.2016.03.001) 27016639

[54] Elsayed GF, Cunningham JP 2017 Structure in neural population recordings: an expected byproduct of simpler phenomena? Nat. Neurosci. 20, 1310–1318. (10.1038/nn.4617) 28783140 PMC5577566

[55] Towlson EK, Vertes PE, Ahnert SE, Schafer WR, Bullmore ET 2013 The rich club of the *C. elegans* neuronal connectome. J. Neurosci. 33, 6380–6387. (10.1523/JNEUROSCI.3784-12.2013) 23575836 PMC4104292

[56] Hums I, Riedl J, Mende F, Kato S, Kaplan HS, Latham R, Sonntag M, Zimmer M 2016 Regulation of two motor patterns enables the gradual adjustment of locomotion strategy in *Caenorhabditis elegans*. eLife 5, 1951 (10.7554/eLife.14116) PMC4880447 27222228

[57] Yan G et al. 2017 Network control principles predict neuron function in the *Caenorhabditis elegans* connectome. Nature 88, 1–17. (10.1038/nature24056) PMC5710776 29045391

[58] Gordus A, Pokala N, Levy S, Flavell SW, Bargmann CI 2015 Feedback from network states generates variability in a probabilistic olfactory circuit. Cell 161, 215–227. (10.1016/j.cell.2015.02.018) 25772698 PMC4821011

[59] Wiltschko AB et al. 2015 Mapping sub-second structure in mouse behavior. Neuron 88, 1121–1135. (10.1016/j.neuron.2015.11.031) 26687221 PMC4708087

[60] Kobak D et al. 2016 Demixed principal component analysis of neural population data. eLife 5, 9424 (10.7554/eLife.10989) PMC4887222 27067378

[61] Fusi S, Miller EK, Rigotti M 2016 Why neurons mix: high dimensionality for higher cognition. Curr. Opin. Neurobiol. 37, 66–74. (10.1016/j.conb.2016.01.010) 26851755

[62] Arous JB, Laffont S, Chatenay D 2009 Molecular and sensory basis of a food related two-state behavior in *C. elegans*. PLoS ONE 4, e7584 (10.1371/journal.pone.0007584) 19851507 PMC2762077

[63] Flavell SW, Pokala N, Macosko EZ, Albrecht DR, Larsch J, Bargmann CI 2013 Serotonin and the neuropeptide PDF initiate and extend opposing behavioral states in *C. elegans*. Cell 154, 1023–1035. (10.1016/j.cell.2013.08.001) 23972393 PMC3942133

[64] Stephens GJ, Johnson-Kerner B, Bialek W, Ryu WS 2008 Dimensionality and dynamics in the behavior of *C. elegans*. PLoS Comput. Biol. 4, e1000028 (10.1371/journal.pcbi.1000028) 18389066 PMC2276863

[65] Liu M, Sharma AK, Shaevitz J, Leifer AM 2018 Temporal processing and context dependency in *C. elegans* response to mechanosensation. eLife 7, e36419 (10.7554/eLife.36419) 29943731 PMC6054533

[66] Ouellette M-H, Desrochers M, Gheta I, Ramos R, Hendricks M 2018 A gate-and-switch model for head orientation behaviors in *C. elegans*. bioRxiv 291005 (10.1101/291005) PMC6325537 30627635

[67] Niell CM, Stryker MP 2010 Modulation of visual responses by behavioral state in mouse visual cortex. Neuron 65, 472–479. (10.1016/j.neuron.2010.01.033) 20188652 PMC3184003

[68] Schneider DM, Nelson A, Mooney R 2014 A synaptic and circuit basis for corollary discharge in the auditory cortex. Nature 513, 189–194. (10.1038/nature13724) 25162524 PMC4248668

[69] Kim AJ, Fitzgerald JK, Maimon G 2015 Cellular evidence for efference copy in *Drosophila* visuomotor processing. Nat. Neurosci. 18, 1247–1255. (10.1038/nn.4083) 26237362 PMC6327952

[70] Fujiwara T, Cruz TL, Bohnslav JP, Chiappe ME 2017 A faithful internal representation of walking movements in the *Drosophila* visual system. Nat. Neurosci. 20, 72–81. (10.1038/nn.4435) 27798632

[71] Cohn R, Morantte I, Ruta V 2015 Coordinated and compartmentalized neuromodulation shapes sensory processing in *Drosophila*. Cell 163, 1742–1755. (10.1016/j.cell.2015.11.019) 26687359 PMC4732734

[72] Leinweber M, Ward DR, Sobczak JM, Attinger A, Keller GB 2017 A sensorimotor circuit in mouse cortex for visual flow predictions. Neuron 95, 1420–1432. (10.1016/j.neuron.2017.08.036) 28910624

[73] Kim AJ, Fenk LM, Lyu C, Maimon G 2017 Quantitative predictions orchestrate visual signaling in *Drosophila*. Cell 168, 280–294. (10.1016/j.cell.2016.12.005) 28065412 PMC6320683

[74] Kawato M 1999 Internal models for motor control and trajectory planning. Curr. Opin Neurobiol. 9, 718–727. (10.1016/S0959-4388(99)00028-8) 10607637

[75] Wolpert DM, Miall RC 1996 Forward models for physiological motor control. Neural Netw. 9, 1265–1279. (10.1016/S0893-6080(96)00035-4) 12662535

[76] Stringer C, Pachitariu M, Steinmetz N et al. 2018 Spontaneous behaviors drive multidimensional, brain-wide population activity. bioRxiv 306019 (10.1101/306019) PMC6525101 31000656

[77] Musall S, Kaufman MT, Gluf S, Churchland AK 2018 Movement-related activity dominates cortex during sensory-guided decision making. bioRxiv 308288 (10.1101/308288)

